# Effects of stress on bone health in children

**DOI:** 10.3389/fendo.2025.1715779

**Published:** 2026-01-07

**Authors:** Zaineb Sohail, Norhayati binti Abd Hadi, Edna Hiu Tung Lam, Muhammad Asghar, Farasat Zaman

**Affiliations:** 1 Faculty of Health Sciences, Universiti Sultan Zainal Abidin – UniSZA, Kuala Terengganu, Malaysia; 2 Department of Life Sciences, School of Science, University of Management and Technology, Lahore, Pakistan; 3 Department of Women’s and Children’s Health, Karolinska Institutet, Stockholm, Sweden; 4 Department of Biomedical Engineering, School of Mechanical and Manufacturing Engineering (SMME), National University of Sciences & Technology (NUST), Islamabad, Pakistan; 5 Department of Biology, Lund University, Lund, Sweden; 6 Department of Sports Sciences and Clinical Biomechanics, University of Southern Denmark, Odense, Denmark; 7 Faculties of Rehabilitation & Allied Health Sciences, Health & Medical Sciences and Pharmaceutical Sciences, Riphah International University, Islamabad, Pakistan; 8 Department of Women's and Children's Health, Biomedicum, Karolinska Institutet, Stockholm, Sweden; 9 Division of Pediatric Endocrinology, Karolinska University Hospital, Stockholm, Sweden

**Keywords:** stress, children, bone, growth, inflammation, diet

## Abstract

Chronic psychological stress is increasingly recognized as a major public health concern, contributing to cardiovascular disease, obesity, asthma, and impaired bone health. Although the mechanisms linking stress to skeletal dysregulation are well characterized in adults, pediatric studies remain limited. Longitudinal and mechanistic studies are needed to clarify how stress affects bone accrual during childhood. Both preclinical and clinical data show that stress can influence bone health through endocrine and immune pathways as well as via altered dietary intake, high or reduced physical activity, medications and disrupted sleep patterns. Elevated stress may also increase oxidative stress, which in turn generates mitochondrial reactive oxygen species (ROS), impairing stem cells differentiation potential, osteoblast and chondrocyte function and suppressing bone formation and growth. In addition, conditions marked by high levels of the pro-inflammatory cytokines TNF-α and IL-6, as well as by elevated exogenous or endogenous glucocorticoids (GCs), further increase cellular oxidative stress. Interventions targeting oxidative stress, such as growth hormone, vitamins C and E, or bisphosphonates, may mitigate skeletal deficits. Here, we review clinical and preclinical evidence on the direct and indirect effects of psychological stress on pediatric bone health.

## 1 Introduction

Stress has emerged as a significant topic in biological sciences and is increasingly studied across diverse fields, including psychology, physiology, and environmental science. According to Lu et al, stress can be defined as a disruption of homeostasis (1). The evolving concept of stress now recognizes both systemic manifestations—mediated through activation of the hypothalamic–pituitary–adrenal (HPA) axis—and localized manifestations arising at specific sites of stressor induction (1, 2).

Chronic psychological stress adversely affects health by inducing lasting physiological, emotional, and behavioral changes that modify disease susceptibility (3, 4). Chronic psychological stress is not only an emerging public health issue but also a risk factor for numerous health disorders, including cardiovascular illnesses, asthma, obesity, and diabetes (4), it can also compromise skeletal integrity via activation of the HPA axis and sympathetic nervous system, low-grade inflammation, and alterations in behavior such as diet, sleep, and physical activity. Chronic stress is known to trigger neuroendocrine changes that result in increased production of ROS (5) both locally and systemically. Elevated ROS levels, in turn, stimulate the release of stress hormones such as cortisol, which may negatively affect multiple tissues, including bone. High levels of ROS have been reported to impair bone health within the musculoskeletal system by inducing undesired apoptosis of bone building cells (osteoblasts and osteocytes), as well as increasing the activity of bone resorbing cells (osteoclasts). Elevated cortisol levels can further disrupt the balance between osteoblast- and osteoclast which is required for bone remodeling. Indeed, epidemiological studies increasingly associate common mental disorders with reduced bone mineral density (BMD) and risks of fractures, highlighting the translational significance of these findings across the lifespan (6–8).

Psychosocial stress in children may originate from various environmental sources, including the home environment, school, neighborhood, peer relationships, and academic pressures. Furthermore, insufficient physical activity and prolonged screen exposure may exacerbate psychosocial stress. Longitudinal studies are warranted to elucidate the associations among psychosocial stress, dietary factors, and bone health. Evidence generated from such research could inform the development of strategies aimed at reducing environmental stressors in children. Mitigating psychosocial stress may, in turn, decrease the need for pharmacological interventions prescribed to alleviate stress or to promote bone health. While the effects of stress on adult health are well-documented, there is a notable gap in our understanding of its specific impact on children’s bone health.

Although very limited data are available on the direct association between stress and bone health in children, we aim to review the existing literature and to connect this evidence with children to identify potential gaps and the need for future research. Specifically, we will examine association between stress, diet, longitudinal bone growth and BMD or bone formation. While numerous pediatric bone disorders have been identified, this review specifically emphasizes stress-related alterations in bone health, with the aim of advancing current understanding of the potential direct and indirect associations between psychosocial stress and bone health in children.

## 2 Methodology

This narrative review was based on a comprehensive literature search conducted in databases such as PubMed/Medline, Web of Science and Google Scholar to identify scientific studies published no earlier than 2020, using keywords such as *“stress, psychological OR psychosocial,” “bone health,” “factors affecting bone health,” “children OR pediatric,”* and *“bone growth.”* Studies were included from age groups such as prepubertal children and adolescents. Studies that assessed responses to stress indicators (e.g., cortisol levels, life events, and chronic conditions) as well as skeletal outcomes (e.g., bone mineral density, growth velocity, and fracture incidence) were included. Owing to the scarcity of data on stress and bone health in children, several studies from other age groups and preclinical findings were also included as supplementary literature to strengthen the evidence regarding any potential association between stress and bone health. Only articles published in English were included, encompassing original preclinical and clinical studies, case reports, and reviews. Furthermore, if any fundamental, mechanistic, or otherwise significant studies related to the manuscript’s keywords were published before 2020, they were included as well. Given that this review employs a narrative design, neither a formal systematic review process nor quantitative analytical procedures were conducted.

## 3 Stress

**Figure 1:**
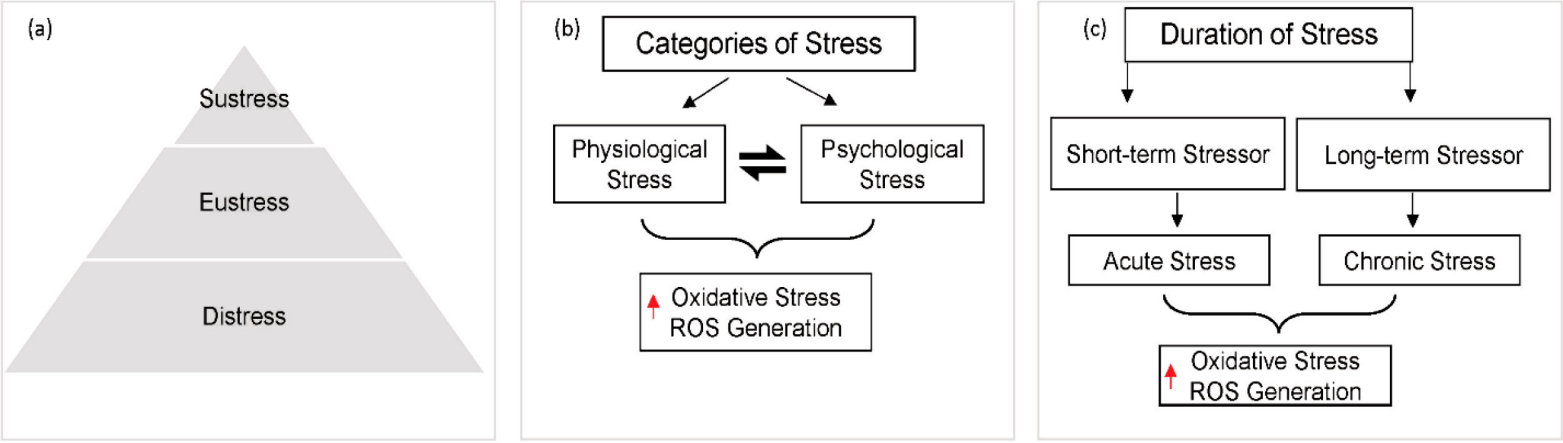
**(a)** Three basic types of stress based on the amount and duration, the little or inadequate levels of stress that do not affect the body are known as sustress. Eustress is the mild form that is positive for the body and helps to perform routine chores while Distress is the high, chronic and persistent level of stress that challenges the body’s hemostasis leading to different diseases and disorders. **(b)** Stress can be categorized in the form of physiological stress or psychological stress. Despite both having different types of stressors, they ultimately trigger oxidative stress in the body. **(c)** Duration of stress, either a short-term stressor leading to acute stress which is transient and momentary, or a long-term stressor leading to chronic stress. Both acute and chronic ultimately disturb the body’s homeostasis by triggering oxidative stress and ROS in the body.

Stress is mediated by a stress system that encompasses a stimulus, various stressors, corresponding physiological and psychological responses, and their subsequent effects. Stress (**Figures 1a–c**) can be classified into three basic types, as illustrated in **Figure 1a**: sustress, eustress, and distress. Sustress is the inadequate amount of stress, while Eustress is mild stress, also known as good stress. Eustress is defined as a temporary, manageable challenge that can improve motivation and adaptation. In contrast to distress, prolonged eustress does not correlate with negative health outcomes (9). However, distress is a chronic or consistent stress condition that challenges homeostasis and is also known as bad stress (10).

Stress is further categorized into physiological and psychological stress (**Figure 1b**) and duration such as acute of chronic stress (**Figure 1c**), which then ultimately leads to oxidative stress in the body. Physiological stress involves the body’s response to direct physical or biological challenges that disturbs the body’s homeostasis (11, 12). Bodily tissues and functions are directly impacted by these stressors, triggering a cascade of physiological responses aimed at restoring balance (13, 14). Physiological stressors (for example, infection, injury, extreme environmental conditions, and metabolic disturbances) trigger systemic responses that include the nervous system, endocrine system, and immune system (15). On the other hand, psychological stress arises when individuals perceive and evaluate events or situations as demanding or threatening to their well-being. This form of stress is inherently subjective and is influenced by an individual’s personality, coping skills, and past experiences (16, 17).

Psychological stress is often initiated by cognitive and emotional stimuli (for example, work or work place related problems, financial strain, relationship issues, sexual dissatisfaction, and perceived threats) and can trigger physiological responses similar to those seen in physiological stress (18–20).

Oxidative stress is characterized by a disruption of the delicate balance between the generation of reactive oxygen species (ROS) and the efficacy of endogenous antioxidant defense mechanisms at the cellular level. Persistent or excessive ROS accumulation can inflict oxidative damage on vital biomolecules, thereby driving the onset and progression of diverse pathological conditions (21, 22). Both physiological and psychological stress can lead to excessive ROS production (23, 24). An increased production of ROS can also occur through various mechanisms, including mitochondrial dysfunction and activation of enzymes like NADPH (Nicotinamide Adenine Dinucleotide Phosphate) oxidase, leading to production of stress hormones and activation of the sympathetic nervous system (25, 26). Simultaneously, chronic psychological stress can impair the body’s antioxidant defense mechanisms, further exacerbating oxidative stress (27). Chronic psychological stress is mechanistically linked to bone loss by sustained ROS, which promotes osteoclastogenesis and compromises osteoblast function (28). The triggers, physiological responses and potential long-term health consequences are all different for both acute and chronic stress. The quick, short-term reaction to a perceived threat or difficulty is known as acute stress (29). It is frequently brought on by distinct, recognizable occurrences, usually of brief duration. The body often returns to its baseline state really fast after the stressful event is over (30). Periodic acute stress is a natural part of life and can even be adaptive, preparing us to confront challenges, but frequent acute stress can be exhausting. The extended and continuous feeling of stress brought on by continuous, long-term stressors is known as chronic stress. Although these stressors might not be as severe on their own as acute stressors, their ongoing nature keeps the body from regaining homeostasis and relaxation. Prolonged stress can have serious negative impacts on one’s physical and emotional well-being (31).

**Figure 2:**
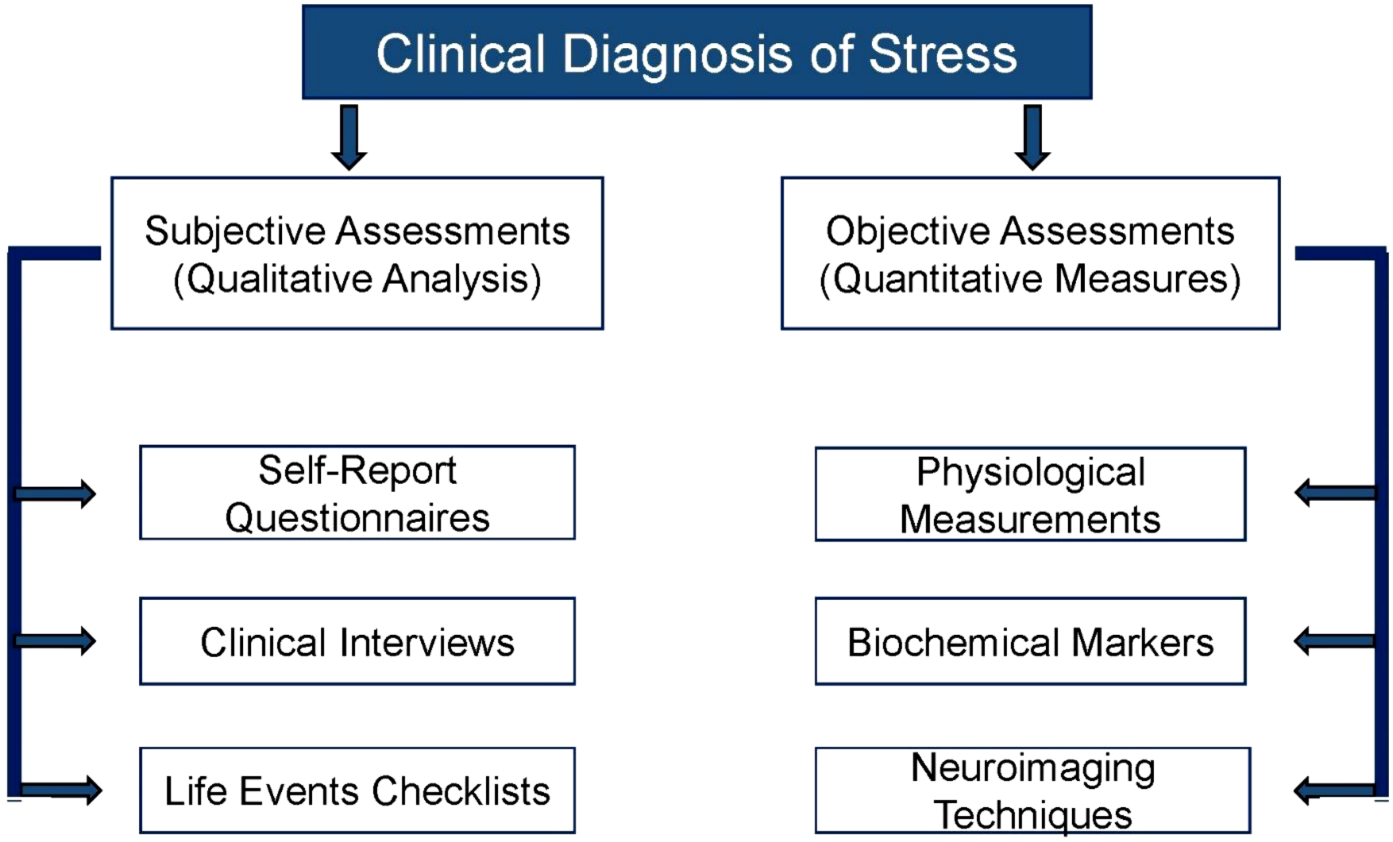
Clinical diagnosis of stress includes both qualitative and quantitative analysis.

Childhood and adolescence represent critical phases of significant bone development; disruptions during these stages can alter the risk of fractures throughout life (32–34). Recent studies emphasize the significance of modifiable early-life exposures, such as built-environment stress buffers, in relation to pediatric BMD (35). Additionally, the introduction of race-neutral pediatric dual-energy X-ray absorptiometry (DXA) norms enhances interpretative accuracy across diverse populations (36). The stress can be measured by using qualitative and quantitative techniques as shown in **Figure 2**.

## 4 Stress-induced ROS production

Chronic stress initiates a series of neuroendocrine changes that result in an increased production of ROS that surpass the body’s antioxidant defenses, thereby causing oxidative stress. Increased oxidative damage has been associated with elevated stress hormone levels, such as cortisol, proposing a connection between psychological stress and tissue oxidative stress in children (37, 38). High levels of ROS can disrupt the normal bones homeostasis in the musculoskeletal system. Oxidative stress induces apoptosis in bone-forming cells (osteoblasts and osteocytes), increases the activity of osteoclasts and inhibits osteogenesis (39). The disturbed bone remodeling results in the imbalance favoring bone loss and the deterioration of bones (40).

## 5 Bone growth

**Figure 3:**
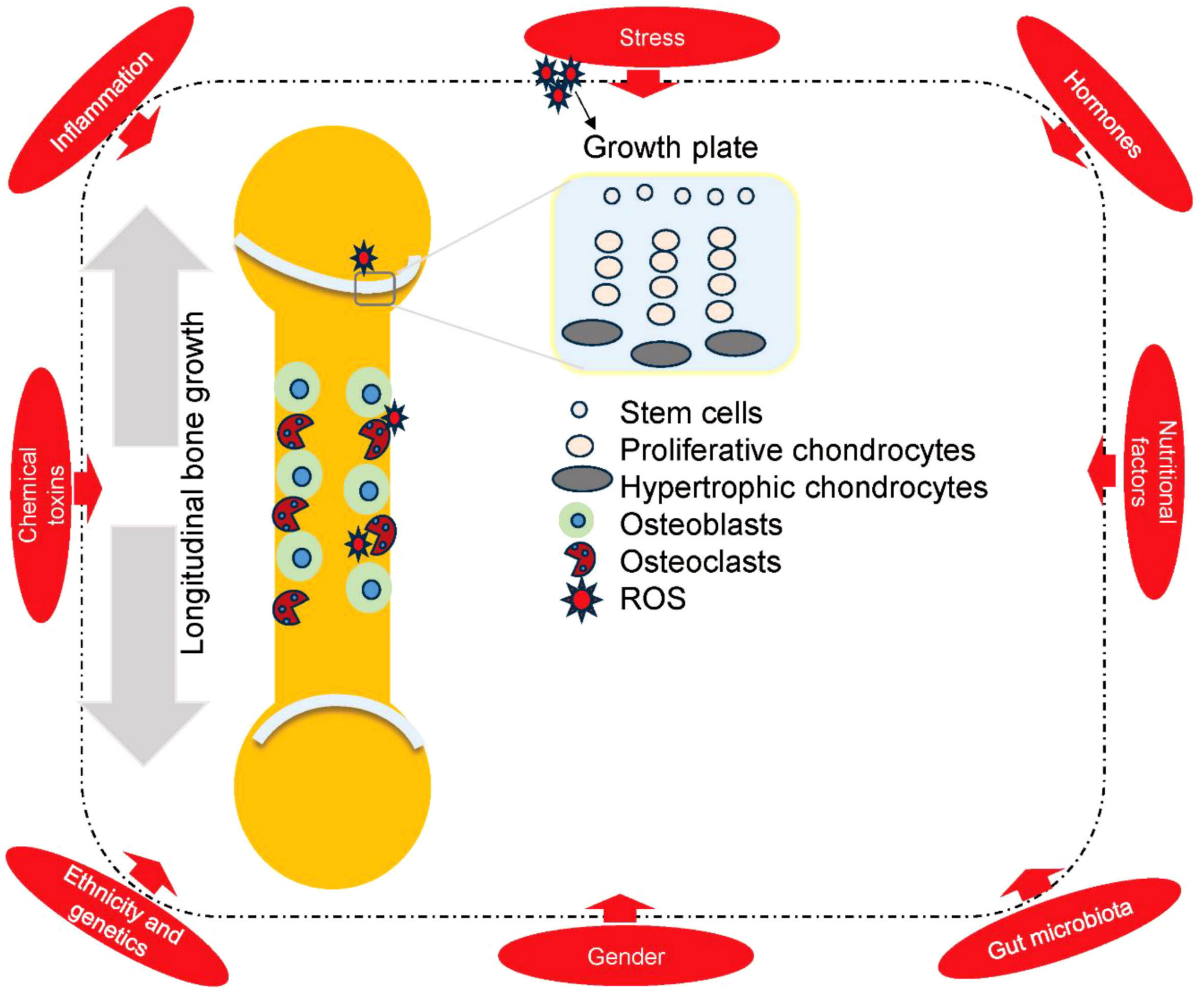
The figure illustrates a long bone, highlighting chondrocytes, osteoblasts, and osteoclasts, along with key factors that disrupt bone formation or growth. Among these, psychological stress which triggers ROS generation is a critical focus of this review. Disturbances in the stem cell, proliferative, or hypertrophic zones of the growth plate impair bone elongation, potentially leading to growth retardation. Likewise, imbalances between osteoblast and osteoclast activity negatively affect bone formation.

Epiphyseal growth plates, also known as physes are present at the ends of long bones (**Figure 3**). Long bones grow vertically due to the activity of these physes in children and adolescents. The growth plate mainly consists of cartilage, which serves as a site for a specialized cell population known as resting or stem cells-like chondrocytes, proliferative chondrocytes and hypertrophic chondrocytes (**Figure 3**). The stem cell zone produces proliferative chondrocytes, forming columnar stacks in the collagenous matrix (41). As the chondrocytes progress towards a hypertrophic state from a resting state, they undergo apoptosis, leaving behind a calcified cartilage scaffold which is then occupied by blood vessels, bone-forming (osteo-progenitor cells), and bone-resorbing (osteoclast precursors) cells. Calcified cartilage is substituted by the bone tissue by the endochondral ossification process, wherein osteoblasts deposit new bone while osteoclasts eliminate remnants of cartilage and immature bone (42). Endocrine signaling, including fluctuations in insulin-like growth factor-1 (IGF-1), growth hormone, and sex hormones, plays a role in this process; growth plate maturation due to the increased estrogen levels during puberty triggers epiphyseal fusion, ultimately leading to linear growth (43, 44).

The dynamic interplay between osteoblasts and osteoclasts helps the bone tissue itself in the modelling and remodeling process. Osteoblasts (originating from the mesenchymal stem cells) produce type I collagen, known as osteoid, which helps in the mineralization to form new bone matrix. Whereas Osteoclasts (monocyte-macrophage lineage multinucleated large cells), on the other hand, get attached to the bone surfaces to resorb the mineralized bone matrix by proteolytic enzymes and acidification (45–47). During growth progression, the bone formation process usually surpasses the bone resorption process, which leads to net bone accrual, thus increasing bone mass till the peak bone mass is achieved in late adolescence (48, 49).

Bone modelling and remodeling are two mechanistically coordinated processes that support bone development (50). Osteoblasts and osteoclasts work on separate surfaces to bring in changes to the size and shape of the bones in reaction to developmental needs and mechanical load; however, modelling plays a vital and exceeding role during growth acceleration (51). On the other hand, remodeling, therefore, is a continuous process in which osteoclasts excavate a little resorption, which is then replenished by osteoblasts with new bone, thus maintaining the strength and mineral balance of the bone (52). In pediatric skeletons, high osteoblastic activity and coordinated osteoclastic resorption at the active growth plate collectively facilitate both longitudinal growth and architectural adaptation of bones (53).

## 6 Stress and longitudinal bone growth in children

Most of the research on stress and bone health focuses on adults and the elderly population. Since infancy and adolescence are the most critical times for bone growth and the times when most peak bone mass is produced, this substantial knowledge gap is especially concerning as it may have an impact on skeletal health throughout one’s lifetime.

In children, there are many factors that are known to be associated with bone health, but there is no such study that depicts the direct association between stress and longitudinal bone growth in children to the best of our knowledge. Preclinical studies showed that endochondral ossification can be disrupted in the growth plates due to chronic stress in mice models, resulting in reduced long-bone length during developmental stages (54). However, several studies have indirectly investigated the association of stress and growth by establishing the link between different factors that raise the levels of stress in family members which ultimately halt the growth of children in the family.

Increased BMI-z is associated with larger bone size/strength by pQCT (peripheral Quantitative Computed tomography) in youth although central adiposity is inversely associated with DXA-BMD (55). After weight loss (e.g., post-bariatric), adolescents may have chronic BMD deficits. Adiposity and bone are site- and compartment-specific across growth (56, 57). In one such study the Youth Life Stress Interview was used to gauge current psychosocial stress in children 7 to 15 years of age in previously institutionalized (PI) youth (58). This study found that PI continued to be thinner and shorter than non-adopted (NA) youth on all three measures. However, in PI kids, the age-and-sex-adjusted BMI rose more quickly. Greater age-and-sex-adjusted BMI was predicted by psychosocial stress during puberty; however, this effect was not group-specific. The study reported that the BMI difference between PI and NA youth narrowed without affecting the height. During puberty, a higher BMI was also linked to higher psychosocial stress.

Children with chronic inflammation not only suffer from growth impairment but also delayed puberty (59, 60). When there is an increase in inflammatory cytokines due to stress in the body, it triggers the release of cortisol by the stimulation of the HPA axis as cortisol represses the inflammation by lowering the production of inflammatory and pro-inflammatory cytokines (61, 62). Meta-analyses demonstrating correlations between perceived stress and increased inflammatory biomarkers demonstrating the clinical importance of such link (63, 64).

Thus, it is evident from the discussion above that inflammation not only affects bone health in children but also alters the endocrine regulation of growth. Higher levels of TNF-α and IL-6 promote osteoclastogenesis and hinder osteoblast differentiation, which shifts the bone turnover towards resorption. So lower levels of inflammatory cytokines are important in order to support healthy and strong bone growth (65, 66).

### 6.1 Stress, bone mineral density and cortical bone thickness

Reduced BMD has been linked to long-term psychological stress via a number of interrelated pathophysiological pathways (67). Chronic stress causes the HPA axis to remain activated, which raises glucocorticoid levels, especially cortisol. This imbalance favors bone resorption over formation by directly impairing osteoblast function and increasing osteoclast activity (68, 69).

In children, studies showed that stress-related endocrine changes, for example, fluctuations in cortisol levels and immune system alterations, can enhance bone loss and hinder bone formation. Observational and experimental evidence suggests that association of high stress with lower bone mass and higher risk of fractures, emphasizing that psychological stress is an evident factor in pediatric bone fragility (70).

Numerous physiological pathways cause chronic stress to have a substantial impact on bone health. One important process is the release of the glucocorticoid hormone – cortisol, which can interfere with bone metabolism if it is continuously secreted. Cortisol can suppress osteoblast activity and encourage bone cell death (71, 72). Additionally, it can affect the synthesis of PTH and reduce calcium absorption, which can result in a negative calcium balance and bone resorption (73, 74).

In addition to cortisol, stress can interfere with the synthesis of sex hormones such as testosterone and estrogen, which are essential for preserving bone density (75–77). Osteoclast activity is increased by this hormonal disruption, especially estrogen deprivation, which speeds up bone loss (77, 78). Stress also causes a persistent low-grade inflammatory state that is marked by a rise in pro-inflammatory cytokines such as TNF-α and IL-6, which promote bone resorption and prevent bone growth (79, 80). Male mice exhibited bone loss via enhanced glucocorticoid signaling in osteoblast and osteocytes and subsequent activation of osteoclasts due to chronic mild stress. However, the skeletal effects of chronic mild stress did not affect female mice (81). Furthermore, pro-inflammatory cytokines such as TNF-α and IL-6, which are produced in response to stress, further impair bone metabolism by promoting osteoclastogenesis (82). These biological mechanisms are compounded by stress-related behavioral changes such as reduced physical activity, poor nutrition, and increased substance use, all of which independently contribute to compromised bone health during life.

In children with ADHD which is a long-term neurodevelopmental disorder, short-term physical exercise helped in reducing the self-reported stress levels while it increased salivary cortisol levels (83), suggesting a cross-talk between stress and ADHD in children. However, studies also show that psychotropic drug usage may also result in low BMD and poor bone health in ADHD children, and the dosage must be prescribed with caution, and examination during the treatment regimen is pivotal (84). In another clinical finding, it was observed that bone fracture risk was increased in ADHD children due to poor bone health as compared to non ADHD children (85). According to a systematic review, it is difficult to conclude the effects of psychostimulant medication on BMD, bone mineral content, and bone turnover in ADHD children; however, it is suggested to acknowledge and not to disregard the association that lies between psychostimulant medications and bone-related parameters (86). Similarly, in another study conducted on ADHD children, those who used stimulants had low DXA measurements of the lumbar spine and femur compared with non-stimulant users (87). Altogether, these clinical findings suggest that children with ADHD are exposed to stress in parallel to psychostimulant medication, which may result in poor bone health.

Cortical bone thickness plays a crucial role in bone strength, which is adversely affected by cortisol levels via a number of processes. In addition to boosting osteoclastogenesis and promoting osteoblast and osteocyte apoptosis, cortisol directly inhibits osteoblast activity and disrupts calcium homeostasis by raising renal calcium excretion and lowering intestinal calcium absorption in individuals (88, 89). These actions compromise the structural integrity of bones by decreasing periosteal bone growth, increasing cortical porosity, and decreasing cortical thickness. Additional mediators between psychological stress and bone loss include β-adrenergic signaling pathways that activate the sympathetic nervous system (90, 91) and psychological stress-related inflammatory cytokines such as IL-6 and TNF-α (92). Thus, chronic stress-induced tailoring in cortical bone can impact overall bone health in children, as high exposure to stress can result in thinner cortical bone, thus increasing the chances of bone fragility and fractures. **Table 1** summarizes the selected cited studies, highlighting the stressors examined, the age groups of the child populations, the skeletal outcomes observed, and any key biochemical or clinical markers reported.

**Table 1:** Summary of selected studies examining the association between stress and bone health in children.

Study (Author, Year)	Stressors investigated	Population (Age)	Key skeletal outcomes	Markers/notes
Zhou et al. (205)	High maternal anxiety/stress in late pregnancy	Infants → Early Childhood	Slower postnatal growth trajectories (length/height)	Maternal self-reported stress; child growth tracking
Simons et al. (208)	Maternal postnatal stress (longitudinal study)	Children (6 years old)	*No direct skeletal measure* (study focus on stress response)	Elevated child cortisol levels (salivary) under maternal stress
Zhong et al. (58)	Psychosocial stress in previously institutionalized (PI) vs. non-adopted youth	Children (7–15 years)	PI youth: lower BMI and height than controls; high stress during puberty predicted greater BMI gains (height unaffected)	Stress measured by Youth Life Stress Interview; anthropometric outcomes (BMI, height)
Ziv-Baran et al. (85)	ADHD diagnosis (chronic stress/behavioral differences)	Children (mean ~10 years)	Increased fracture risk in children with ADHD vs. non-ADHD	No direct biomarker; fracture incidence compared between cohorts
Feuer et al. (87)	Stimulant medication use (ADHD treatment)	Children & Adolescents	Lower bone mass (DXA BMD at spine and femur) in stimulant users vs. non-users	Clinical DXA measurements of BMD; suggests medication as a stressor to bone metabolism
Lempesis, I.G., et al. (130)	GC usage in children with JIA	155 children	25.8% exhibited growth delay	Prolonged disease duration, reduced physical activity, GC usage
Pan, F., et al. (139) Tang, Y., et al., 2022	Sleep	Population-based studies	Compromised bone health and reduced BMD	No direct biomarker, sleep schedule and sleep duration.
Jain, C., et al. (140)	GCs usage	Children and adolescents	significantly contribute to secondary osteoporosis	GCs, antiepileptics, and psychiatric medications including stimulants and antipsychotics
Zhang, J., et al. (163)	Increased sedentary time (Low physical activity)	Childhood and adolescence	Effecting both mental health and skeletal development	Suggests a bidirectional cycle wherein stress and internalizing issues foster inactivity, potentially undermining both mental health and skeletal development
Chang L., et al., 2024	Moderate-to-vigorous physical activity (MVPA)	2–15 years old children	Positive correlation with heel bone BMD and bone mineral apparent density	dietary calcium and vitamin D intake were not predictive, emphasizing the predominance of physical activity over diet in this age group

The table details the types of stressors investigated, the age ranges of the pediatric populations studied, the observed skeletal outcomes, and any relevant biochemical, clinical, or molecular markers reported. This synthesis highlights both the direct and indirect pathways through which stress may influence pediatric bone development.

## 7 Direct crosstalk between stress and bone health

### 7.1 Glucocorticoids, oxidative stress and bone health

High levels of endogenous/exogenous glucocorticoids (GCs) also suppress bone growth and bone formation by triggering ROS-mediated oxidative stress (93). In Cushing’s syndrome, where endogenous GC levels are abnormally elevated, deleterious effects on bone metabolism have been observed. These include decreased osteoblast activity, reduced BMD and increased apoptosis of both osteoblasts and osteoclasts, ultimately leading to a higher risk of bone fractures (94).

In pediatric patients with juvenile idiopathic arthritis (JIA), both inflammation and GC use contribute to impaired growth and bone formation. Although tumor necrosis factor-alpha (TNF-α) antagonists such as etanercept demonstrate limited efficacy in systemic JIA, growth hormone (GH) therapy has proven effective in enhancing growth velocity. Prompt initiation of GH therapy may thus help preserve normal growth patterns and mitigate long-term height reduction or growth deficit. Preclinical studies showed that treatment with GCs prevents longitudinal bone growth by inducing undesired apoptosis of chondrocytes within the growth plates (95). Following GC withdrawal, growth recovery remains limited; however, exogenous administration of GH restores growth plate function by improving chondrocytes activity, and promoting bone elongation (96). Inhaled GC therapy in prepubertal children with asthma has also been associated with a reduction in early growth velocity, particularly during the first year of treatment, regardless of dosage. While asthma alone does not impede growth, more severe cases combined with prolonged GC exposure have been linked to significant growth retardation (97), emphasizing the importance of individualized growth monitoring. Interestingly, GH has also been reported to reduce oxidative stress in cells (98). These findings are consistent with previous studies demonstrating that GH therapy alleviates GC-induced growth retardation in children with JIA. Dexamethasone administration has been shown to markedly increase apoptosis in both hypertrophic and proliferative chondrocytes within rat growth plates, resulting in impaired bone development. This outcome correlates with elevated caspase-3 levels and decreased expression of anti-apoptotic proteins Bcl-2, Bcl-x and Parathyroid hormone-related protein (PTHrP), suggesting that GC-induced chondrocyte apoptosis plays a crucial role when causing growth retardation (99). Prolonged GC therapy in children, particularly at high doses or during early developmental stages, may also lead to irreversible growth retardation. Although catch-up growth may occur after short-term exposure, extended treatment - such as in nephrotic syndrome or post-transplant management - often results in persistent impairment due to disrupted cellular metabolism, inhibition of RNAs and protein synthesis, and reduced cell proliferation and differentiation (100).

In a Duchenne muscular dystrophy (DMD) murine model, GC therapy impaired somatic growth and skeletal integrity while improving muscle strength. Co-administration of GH and insulin-like growth factor 1 (IGF-1) mitigated growth retardation and enhanced muscular strength; however, it did not improve bone microarchitecture or turnover markers (101), underscoring the need for further research before clinical implementation. In another murine model, dexamethasone administration suppressed longitudinal bone growth; however, this effect was largely prevented in transgenic animals with elevated C-type natriuretic peptide (CNP) expression, which restored bone elongation and growth plate architecture (102). Bisphosphonates have also been reported to improve bone health in children with DMD (103), and low doses of alendronate have additionally been shown to reduce oxidative stress in ligament stem cells (104). These findings highlight the therapeutic potential of GH, bisphosphonates, and CNP in mitigating GC-induced skeletal growth deficits in pediatric populations, where they are reported to act in part by reducing oxidative stress alongside other mechanisms.

Altogether, these findings suggest that although the use of GCs effectively suppresses inflammation, it may also trigger undesired ROS generation, which directly or indirectly can negatively affect various tissues, including bone, thereby impairing growth and bone formation in growing individuals. Treatment with GH reduces cellular oxidative stress, suggesting the possibility of preventive strategies.

## 8 Indirect crosstalk between stress and bone health

### 8.1 Stress-induced changes in diet

In children, nutritional choices and dietary patterns are suggestively influenced by psychological stress as higher stress levels provoke poorer diet. Recent cross-sectional findings showed that the intake of fresh fruits, vegetables, whole grains, and lean proteins was significantly lower in the stressed youth (105). Children under stress are more prone to eating unhealthy and junk food, including fatty, sugary or salty snacks in lieu of nutritional food. A lot of studies have shown the effect of stress on food intake. For example a study showed that stress induced loss of appetite in 40% of the participants, 40% participants reported increased appetite and 20% reported no change in the appetite (106). Stress tends to change the hormones that are responsible for appetite and perhaps this is the reason for the different eating patterns of a person when exposed to stress. Usually with acute stress the sympathetic-adrenal-medullary system gets activated and consequently starts releasing catecholamines (adrenaline & noradrenaline) (107, 108). Noradrenaline is known as a suppressor of appetite during acute stress (109, 110). Chronic stress, on the other hand, hyperactivates the HPA axis that results in the production of CRH, adrenocorticotropic hormone (ACTH), and glucocorticoids (cortisol) (111, 112). CRH is responsible for lowering appetite in humans (113, 114). Leptin which is also known as the “Satiety Hormone” exerts an anorectic effect by increasing the expression of CRH in the hypothalamus. On the other hand, CRH helps in the release of cortisol from adrenal glands downstream (115, 116). During conditions of stress, the final product of the HPA axis are glucocorticoids (cortisol) which are orexigenic (117, 118). Glucocorticoids also stimulate other orexigenic peptides and inhibit CRH expression (119–121). Furthermore, it is important to note that glucocorticoids are orexigenic only in non-stressed states of the body whilst they act as strong anorexigenic substances in stressed states (122).

Stress significantly influences dietary patterns across all ages, with some common trends and age-specific nuances. Primary caregivers in infancy regulate food patterns, whilst feeding behaviors can be disrupted by babies stress, which is frequently caused by mother’s stress or inconsistent caregiving (123). As children get older, stress from family or school are linked to a change in eating habits, which includes consuming more sugary and fatty meals and fewer fruits and vegetables (124, 125). This could be the result of utilizing emotional eating as a coping strategy. Teenagers are especially susceptible to this, and social and academic pressures can further lead to disordered eating practices and excessive snacking (126, 127).

In addition to disrupting circadian eating patterns, stress-induced shifts toward ultra-processed, high-sugar, and high-fat eating patterns can impair calcium and protein adequacy and alter the gut flora- pathways increasingly implicated in bone loss (128). Through the release of pro-inflammatory cytokines, stress-induced changes in the makeup of the gut microbiome causes dysbiosis and increased intestinal permeability, which in turn fuels systemic inflammation (129). Glucocorticoid-mediated immunosuppression causes notable functional degradation of the immune system, which is reflected in decreased natural killer cell activity, decreased lymphocyte proliferation, and changed cytokine production patterns that transition from Th1 to Th2 cells response (130).

**Figure 4:**
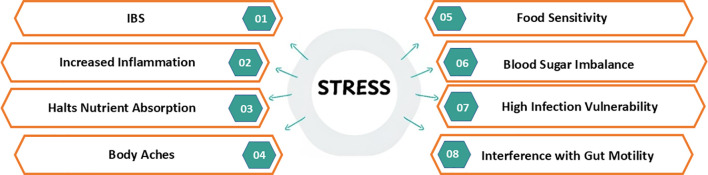
This Figure shows an association between stress, dietary patterns and immunity. By affecting dietary patterns, it may lead to Irritable Bowel Disease (IBD), interfere with gut motility increased inflammation, halt nutrient absorption, cause body aches, may lead to food sensitivities, disturb blood sugar balance due to high sugary food intake, low immunity leading to high infection risk.

The combination of immune compromise and digestive dysfunction leads to a self-reinforcing cycle in which compromised gut barrier function increases exposure to antigens, which in turn triggers inflammatory cascades and intensifies stress responses (**Figure 4**). This ultimately explains why chronically stressed populations are more susceptible to autoimmune conditions, infectious diseases, and gastrointestinal disorders, and several food sensitivities (131, 132). Diets that place an emphasis on fruits and vegetables, dairy products, and overall quality (such as DASH or Mediterranean-like diets) are associated with balanced gut microbiota, good immunity, better bone mineral density and a lower risk of fracture (133–135). This suggests that nutritional imbalance caused by chronic stress can directly or indirectly impair bone health, as chronic stress limits the intake of a protein/calcium-rich diet, increases inflammation, and interferes with other lifestyle risks- ultimately impacting children’s bone health and skeletal growth.

### 8.2 Stress, sleep disorder and bone health

Sleep plays a crucial role in maintaining normal endocrine function and supporting nocturnal bone remodeling, whereas sleep disturbances stemming from psychological stress can compromise bone health (136). Stress-induced insomnia, as well as delayed or disrupted sleep, adversely affects hormonal regulation, leading to decreased growth hormone and melatonin levels and elevated nocturnal cortisol, thereby hindering bone growth and formation. (137). Disturbed sleep can also trigger oxidative stress and ROS generation (138), which in turn negatively affect multiple tissues, including bone. Short naps or irregular sleep duration have been associated with reduced BMD in population-based studies, suggesting that inadequate sleep may compromise bone health during critical developmental periods (139, 140). Moreover, sleep disturbances induce systemic inflammation and sympathetic activation, both of which adversely impact osteoblast function and accelerate osteoclast activity (141). These findings suggest that stress-related sleep disturbances during childhood and adolescence may hinder the attainment of peak bone mass, although dedicated pediatric studies remain limited.

### 8.3 Medications in childhood mental health disorders, stress and bone health

Children with mental health disorders often require prolonged pharmacotherapy during periods of rapid skeletal development; however, several commonly used drug classes are now recognized to affect BMD, growth velocity, and fracture risk. A recent review on pediatric bone health highlights that GCs, antiepileptics, and psychiatric medications—including stimulants and antipsychotics—significantly contribute to secondary osteoporosis in children and adolescents (142, 143). Prolonged and high-dose use of these medications may also increase oxidative stress and ROS generation, in addition to causing systemic effects through hormonal imbalances.

Attention-Deficit/Hyperactivity Disorder (ADHD) is a condition that affects an individual’s ability to focus, control impulses, and regulate activity levels. Stimulant medications, such as methylphenidate and amphetamine derivatives, remain the first-line treatment, whereas non-stimulants (atomoxetine, guanfacine, clonidine) are prescribed when stimulants are not tolerated; both classes act primarily by enhancing catecholaminergic neurotransmission. Analysis of the US NHANES cohort of 8–20-year-olds revealed that stimulant users had significantly lower total-body and lumbar spine BMD and BMC than non-users, particularly among males (144). *In vitro* studies further indicate that psychostimulants—including atomoxetine, modafinil, and guanfacine—impair the osteogenic differentiation and migration of mesenchymal stem cells at therapeutic concentrations, suggesting a potential direct detrimental effect on osteoblast function (145). Atomoxetine has been reported to induce oxidative stress and ROS generation at higher concentrations in human neuron-like cells (146). These findings suggest that if similar mechanisms operate in osteoblasts or chondrocytes, they may likewise trigger adverse effects on bone formation and growth. Anti-seizure and antiepileptic drugs (AEDs) are also linked to poor bone health, as prolonged AED administration appears to significantly reduce BMD (147). Children receiving long-term antiepileptic therapy exhibited markedly lower bone mineral values and an increased risk of falls and fractures, highlighting the need for monitoring and preventive interventions (148). Similarly, evidence regarding antipsychotics and bone fragility indicates that prolactin-raising agents (e.g., risperidone, paliperidone) reduce BMD and elevate fracture risk, particularly with prolonged exposure (149). Population-based case–control studies show that antipsychotic use correlates with higher rates of osteoporotic fractures (150).

In summary, these findings support the routine assessment of medication exposure when evaluating bone outcomes in children under stress, alongside the implementation of bone-protective strategies—such as optimized nutrition, vitamin D supplementation, and weight-bearing exercise—for those receiving long-term psychotropic or steroid therapy (151). In addition, therapeutic approaches aimed at reducing oxidative stress or ROS may further enhance treatment outcomes.

### 8.4 Physical activity

Physical exercise can also act as a physiological stressor and exert direct effects on bone metabolism through mechanical loading and hormonal regulation. Physical activity leads to the release of GCs into circulation; short-term elevations in GCs can have beneficial effects on multiple tissues, including bone. However, prolonged or intense exercise may result in hypercortisolism, which in turn can increase the risk of osteoporosis (152).

### 8.5 High-intensity exercise, oxidative stress and bone health

High-intensity and repetitive exercise influence bone remodeling, in part, via oxidative stress mechanisms, with new findings indicating that excessive high-intensity training generates elevated amounts of ROS that may disrupt bone homeostasis (153). Moderate exercise enhances antioxidant defenses through hormesis; however, severe or prolonged high-intensity exercise can overwhelm antioxidant mechanisms and intensify oxidative stress (154). Prolonged exposure to ROS may disturb the equilibrium between osteoblast and osteoclast and enhance inflammatory signaling, leading to cellular senescence and skeletal disorders when antioxidant defenses are overwhelmed (155, 156). Appropriately dosed impact and resistance exercises promote the bone accrual in growing individuals, whereas excessively high training loads without sufficient recovery may theoretically diminish skeletal benefits due to cumulative oxidative stress, highlighting a non-linear (U-shaped) relationship between exercise intensity and bone health (157). Altogether, these findings also indicate a role of oxidative stress in high-intensity exercises.

### 8.6 Low physical inactivity, oxidative stress and bone health

Sedentary behavior is well recognized as a risk factor for skeletal health during adolescence. Indeed, physical inactivity has been shown to increase cellular oxidative stress (158), indicating an active crosstalk between physical activity and bone growth. A systematic review demonstrated that prolonged sedentary time is associated with reduced bone mass, impaired microstructure, and diminished bone strength in children, adolescents, and young adults (159). School-based physical education appears to confer protective effects on bone health (160). In children aged 2 to 15 years, moderate-to-vigorous physical activity (MVPA) was positively correlated with heel bone BMD and bone mineral apparent density, whereas dietary calcium and vitamin D intake were not predictive, emphasizing the predominance of physical activity over diet in this age group (161). The World Health Organization’s latest guidelines also recommend that children and adolescents engage in at least 60 minutes of MVPA daily and limit sedentary screen time to support healthy musculoskeletal development. Concurrently, mental health data indicate that insufficient physical activity and excessive sedentary behavior are associated with an increased likelihood of depressive symptoms in adolescents, suggesting that psychological distress often coincides with a transition to more sedentary, low-activity lifestyles (162). Longitudinal meta-analysis reveals that increased sedentary time in childhood and adolescence forecasts subsequent depression and anxiety, suggesting a bidirectional cycle wherein stress and internalizing issues foster inactivity, potentially undermining both mental health and skeletal development (163). Moderate-intensity exercise improves anxiety, depression, and stress scores, thereby reinforcing the notion that disrupting the stress-inactivity cycle can concurrently enhance psychological well-being and bone health (164).

## 9 Stress and oxidative stress in pediatric bone disorders

Stress-induced ROS can damage bone tissue through multiple mechanisms, and one well-studied mechanism is the disruption of the balance between osteoblasts and osteoclasts. Chronic stress elevates ROS levels, which in turn induce undesired cell death not only in chondrocytes but also in osteoblasts and osteocytes, while simultaneously promoting osteoclast activity; together, these effects lead to impaired growth, decreased BMD, and an increased risk of fractures. Oxidative stress can also trigger bone aging by disrupting the redox equilibrium and compromising the functionality of bone cells. Given that ROS contribute to skeletal degeneration, targeting redox pathways may offer promising treatment methods for age-related bone issues (165); however, further study is necessary to optimize these approaches. Below are several examples of pediatric bone and musculoskeletal diseases in which stress-induced ROS has been reported to play a central role.

### 9.1 Joint pain

Psychological or inflammatory stress can increase ROS levels in bone joints, leading to inflammation and cartilage deterioration, and ultimately exacerbating pain (166). In juvenile idiopathic arthritis, an imbalance between ROS production and neutralization results in oxidative stress within the joints (167). This oxidative imbalance is associated with synovial inflammation and accelerated joint degeneration. Consequently, the body’s antioxidant defenses become compromised and fail to counteract ROS-induced damage, further worsening joint pain in affected children.

### 9.2 Bone deformities

Excessive ROS can impair normal bone development and compromise structural integrity, potentially resulting in skeletal abnormalities (168). Kashin–Beck disease is a notable example of an endemic pediatric osteoarthropathy in which oxidative stress plays a critical pathogenic role (169). ROS-induced stress leads to chondrocyte necrosis in the growth plates, causing impaired endochondral ossification and subsequent bone and joint deformities (170). This underscores that persistent oxidative stress can disrupt developmental processes and damage cartilage and bone cells, ultimately leading to long-lasting structural defects. Desmosterolosis, a disorder caused by mutations in DHCR24 (3β-hydroxysterol-Δ24 reductase), also impairs bone elongation by increasing oxidative stress in chondrocytes. Under normal conditions, DHCR24 functions as a ROS scavenger; its absence results in reduced proliferation and differentiation such as premature hypertrophy of growth plate chondrocytes—effects that can be reversed through antioxidant (171). These findings illustrate how ROS disrupts chondrocyte function and normal bone growth and formation.

### 9.3 Fetal disabilities

A preclinical study showed that pregnant mice exposed to lipopolysaccharide (LPS) experienced intrauterine fetal death (IUFD), growth retardation, and impaired skeletal development, effects that were associated with elevated levels of ROS and tumor necrosis factor-α (TNF-α) (172). This study also demonstrated that pretreatment with the antioxidant N-acetylcysteine (NAC) markedly enhanced fetal survival and osseous development, whereas posttreatment did not alleviate oxidative damage and exacerbated results. Another similar preclinical study showed that maternal exposure to LPS resulted in embryonic mortality, growth limitation, and delayed skeletal ossification, associated with elevated ROS and oxidative damage. Pretreatment with ascorbic acid markedly decreased fetal mortality and enhanced bone formation by alleviating oxidative stress, whereas post-treatment was less efficacious in averting IUFD (173). Also, post-treatment with melatonin decreased IUFD, while a combination of pre- and post-treatment nearly eradicated fetal loss and enhanced skeletal results by mitigating oxidative stress (174). In a rat model of diabetic pregnancy, maternal hyperglycemia resulted in embryonic resorption, growth limitation, and skeletal malformations, consequences associated with increased ROS. Supplementation with the antioxidant Lipoic acid markedly diminished these defects without affecting blood glucose levels, indicating that oxidative stress is a crucial factor in diabetic embryopathy and compromised skeletal development (175).

Altogether, these findings suggest that ROS generation negatively affects multiple organs, and its inhibition may prevent these adverse effects.

### 9.4 Short stature

Chronic oxidative stress may impede longitudinal bone growth, leading to reduced height (176). Excess ROS in the growth plate microenvironment can trigger chondrocyte death and impede cartilage matrix formation (177). This oxidative damage impairs endochondral ossification at the growth plate, potentially hindering the rate of bone elongation (178). Children experiencing significant psychosocial stress, resulting in increased cortisol and ROS, frequently exhibit growth retardation, which aligns with the ROS-induced impairment of growth plate function (179).

In mice, exposure to the cyanobacterial toxin microcystin-LR (MC-LR) increased ROS accumulation in bone marrow, resulting in the senescence of bone marrow mesenchymal stem cells (BMSCs) and hindered bone growth (180). Further, this alteration diminished osteogenic capacity and impaired glutamine metabolism through the Hippo pathway, ultimately leading to trabecular bone loss and retarded skeletal growth. Radial shockwaves treatment which promotes bone growth (181), has also been shown to reduce oxidative stress (182).

### 9.5 Dental abnormalities

The oral environment of children is also susceptible to oxidative stress, which can lead to damage to tooth tissue (183). In severe early childhood dental caries, an imbalance favoring ROS results in damage to lipids, proteins, and DNA within the tooth structure, thereby compromising enamel integrity (184–186). Oxidative stress in saliva generates a pro-inflammatory environment that fosters cariogenic bacterial proliferation and accelerates the tissue degradation (187). Consequently, increased ROS may lead to enamel hypomineralization and other dental anomalies (188); however, the direct connections to psychosocial stress require further elucidation.

### 9.6 Muscular hypotonia or laxity

Excessive ROS can damage muscle fibers and connective tissue, potentially leading to decreased muscle tone or joint laxity (189, 190). Oxidative stress in muscle cells impairs mitochondrial function, leading to protein degradation and ultimately diminishing muscular contractions over time (191). In childhood neuromuscular illnesses such as Duchenne muscular dystrophy, oxidative stress is a primary factor in muscle fiber degradation and weakening (192). The limited research on hypotonia in otherwise healthy, stressed children suggests that ROS may impair muscle physiology, leading to reduced muscle tone and stability due to oxidative damage.

### 9.7 Osteomyelitis

Osteomyelitis involves severe inflammation in which elevated ROS levels function as a double-edged sword: on the one hand, they contribute to bacterial elimination, while on the other hand, they simultaneously promote bone destruction. Chronic osteomyelitis, in particular, results in a sustained state of oxidative stress within bone tissue, exacerbating inflammatory damage and impairing tissue regeneration (193). Immune cells of the body produce ROS to fight infectious agents; however, increased levels of ROS can also deteriorate the bone matrix and surrounding healthy tissue, resulting in osteolysis and impaired recovery (194, 195). Individuals with chronic osteomyelitis show a disrupted oxidant-antioxidant equilibrium, suggesting that ROS overproduction plays a pivotal role in the pathogenesis of this pediatric bone infection.

### 9.8 Osteogenesis imperfecta

Osteogenesis imperfecta (OI) is primarily a hereditary collagen mutation disorder; however, elevated ROS levels have also been associated with its pathogenesis. Misfolded collagen in OI induces endoplasmic reticulum stress in osteoblasts, consequently increasing intracellular ROS (196). The resultant oxidative stress further halts collagen processing and bone matrix formation, potentially aggravating bone fragility (197). This feed-forward loop, where mutant collagen causes ROS and ROS compromises collagen quality, demonstrates that oxidative stress may exacerbate the skeletal abnormalities in OI, even if stress-induced ROS is not the primary cause (198).

### 9.9 Rickets

Rickets which typically results from deficiencies in vitamin D or calcium, has also been linked to oxidative stress (199). Children suffering from dietary rickets exhibit elevated indicators of oxidative damage and reduced antioxidant defenses (200). In this context, elevated ROS levels may impair mineralization in growth-plate cartilage by damaging the cellular and extracellular components essential for calcification. Vitamin D repletion diminish oxidative stress in rickets, indicating that some aspects of skeletal disease (bone softness and deformity) may be indirectly exacerbated by an elevated oxidative state (201). Oxidative stress can also trigger bone aging by disrupting the redox equilibrium and compromising the functionality of bone cells. Given that ROS contribute to skeletal degeneration, targeting redox pathways may offer promising treatment methods for age-related bone issues; however, further study is necessary to optimize these approaches.

## 10 Psychosocial environment and growth in children

In children, psychosocial growth retardation, also referred to as psychosocial dwarfism or psychosocial short stature, is a syndrome in which emotional deprivation and chronic stress may result in deficiency of GH along with impaired somatic growth and behavioral disturbances. Emotionally supportive environments play a crucial role in the normal growth of a child, as psychosocial deprivation can disturb growth and trigger other growth disorders (202), including delayed puberty (203). Psychosocial short stature is a growth disorder associated with exposure to an abusive or adverse environment. Clinically, it presents as either short stature or growth failure in the presence of a normal BMI and an increased appetite. In this condition, although the children are GH deficient, the growth velocity may improve in a supportive environment (204). In summary, psychosocial factors should be given major consideration in the management of short stature and pubertal delay.

## 11 Maternal stress and bone health

In children, early life stress, particularly prenatal maternal stress, has been associated with enduring impacts on skeletal development. Prenatal stress can disrupt fetal bone development, with the timing and nature of the stress affecting the severity of bone abnormalities. A cohort study reported that infants of mothers who experienced high stress and anxiety in their last trimesters, showed poorer growth trajectories not only in infancy but also in early childhood (205).

Early life stress (ELS) and pre-natal maternal stress (PMS) have a great impact on later stage behaviors of children and the psyche of an individual and may lead to neurodegenerative disorders (206). Adverse events and exposures may occur during fetal life, like a mother’s undernutrition, complicated pregnancy-specific stressors or any other factors that are stressful to the expecting mother, such as neglect, social violence, parental loss, all these factors come under ELS whereas physical or sexual abuse, exposure to terrorism, war or natural disasters like earthquakes, to the expecting mother is known as PMS (207). However, in one longitudinal study, a strong relationship was found between maternal postnatal stress and her child’s high cortisol levels at the age of 6 years (208). Whereas in another study, maternal depression symptoms do not consistently increase offspring cortisol biomarkers across cohorts, suggesting complicated buffering of fetal stress exposure (209). Strong human linkages from prenatal stress to child BMD have yet to be proven to illustrate a pivotal research gap.

Preclinical studies in animals indicate that prenatal stress adversely affects offspring bone mass and remodeling; however, human data directly associating prenatal stress with offspring bone mineral density are limited and inconsistent, highlighting a significant research gap (210, 211). Thus, early life stressful exposures to the child as early as the womb, can impact the child’s bone health.

## 12 Conclusion

Psychological stress adversely affects bone health across the lifespan, impairing longitudinal growth, microarchitecture, BMD, and turnover, largely via ROS-mediated disruption of osteoblast, chondrocyte, and stem cells. Future research should prioritize longitudinal studies from early childhood, with attention to sex-specific effects and mechanistic pathways. Understanding stress-related bone impairment in children is critical for developing early-life interventions and informing policies to reduce environmental stressors. Notably, no studies have directly examined these associations in healthy, typically developing children, highlighting a key gap for guiding strategies to preserve skeletal health across development.
